# Combined Transcriptomics and Metabolomics Analyses in Grass Carp Under Anesthetic Stress

**DOI:** 10.3389/fcimb.2022.931696

**Published:** 2022-07-11

**Authors:** Tianwei Wang, Yali Wang, Xueting Liu, Xiaoning Gao, Kun Hu

**Affiliations:** ^1^National Pathogen Collection Center for Aquatic Animals, Shanghai Engineering Research Center of Aquaculture, National Demonstration Center for Experimental Fisheries Science Education, Shanghai Ocean University, Shanghai, China; ^2^National Fisheries Technical Extension Center, Ministry of Rural Agriculture, Beijing, China; ^3^Key Laboratory of East China Sea Fishery Resources Exploitation, Ministry of Agriculture, East China Sea Fisheries Research Institute, Chinese Academy of FisherySciences, Shanghai, China

**Keywords:** anesthetics, ecotoxicology, transcriptome, metabolome, arachidonic acid

## Abstract

*Ctenopharyngodon Idella*, as a common freshwater bony fish, is more susceptible to various diseases than other carp species, so it has been proposed as a test organism for toxicological analysis In this study, *C. idella* were anesthetized with MS-222 and 2-PE, and the related anesthetic mechanism and toxic effects were revealed by transcriptomics and metabolomics analyses. When the concentration of MS-222 was 80 mg/L and 200 mg/L, 179 and 887 differentially expressed genes (DEGs), respectively, were identified in the brain tissue of *C. idella*. When the concentration of 2-PE was 0.6 mL/L and 1.2 mL/L, 498 and 514 DEGs were identified. The DEGs associated with MS-222 treatment were enriched in immune pathways, lipid metabolism, amino acid metabolism, and various signaling pathways; DEGs associated with 2-PE treatment were enriched in immunity and amino acid metabolism. In total, 304 metabolites were identified using a combination of positive and negative ion modes in mass spectrometry. The common differential metabolites identified in the MS-222 high and low concentration groups were 20-HETE and 12(R)-HETE; the common significant differential metabolite identified in the 2-PE high and low concentration groups was salidroside. In combination with the transcriptomics analysis and metabolomics analysis, the results showed that with the MS-222 and 2-PE concentrations used in this experiment, the metabolism of arachidonic acid in *C. idella* was inhibited by MS-222, and 2-PE affected the upstream and downstream metabolic pathways of arachidonic acid metabolism, thereby affecting the metabolism of arachidonic acid. Both anesthetics induce sedation by affecting related metabolites that affect stress response and autoimmunity. Metabolomics results showed that neither anesthetic had a significant effect on cortisol expression.

## 1 Introduction

Fresh fish consumption is a unique consumption pattern in China, and the market and influence are huge. The transportation of live fish often leads to a sudden change in the living environment of fish, which leads to a stress response in fish. Compared with the normal state, the physiological and biochemical processes such as hormone secretion and material and energy metabolism in fish change significantly under stress, which directly affects the health of fish and even threatens life and adversely affects the quality of their muscles (Xiaopeng et al., 2021).

Because anesthetics have a good sedative effect, they have been widely used in live fish transportation in recent years (Peng et al., 2021). The rational use of anesthetics alleviates the pain of fish, induces sedation, and reduces the mechanical damage to fish during the process (Wenhao et al., 2020). It also lowers the metabolism of fish, delays the deterioration of water quality, and reduces the respiratory rate of fish, which helps them to adapt to hypoxia stress during long-term transportation. At present, there are more than 30 anesthetics commonly used in fish used in aquatic products, of which 2-phenoxyethanol (2-PE) (Ortuno et al., 2002; Inoue et al., 2004; Vaughan et al., 2008), MS-222 (Matsche, 2011; Rozynski et al., 2018), clove oil (Hekimoglu et al., 2017; Pattanasiri et al., 2017), and carbon dioxide are the most commonly used (Xie and Cao, 2021).

MS-222, also known as tricaine methanesulfonate or tricaine mesylate, is one of the most commonly used anesthetics in aquaculture production practice. MS-222 has a rapid effect on aquatic animals and exhibits short recovery time and no toxicity to humans. It is the only FDA-approved narcotic for fish consumption (Yatao et al., 2019). 2-PE, also known as ethylene glycol phenyl ether (Territory and Xiaoyan, 2016) is also a commonly used anesthetic in fish. The exact mechanism of its action in fish remains unclear. However, it may involve the expansion of nerve cell membrane (Burka et al., 1997) and the synthesis and metabolism of hormones that inhibit the central nervous system. Because 2-PE has a safe range of administration, it reduces animal mortality (Toni et al., 2015). 2-PE anesthetizes animals quickly, the recovery time is short, and the side effects are small. Narcotics are widely used in aquaculture, but the mechanisms and toxic effects of narcotics on fish remain unclear.

*Ctenopharyngodon Idella*, a representative teleost fish, is the largest freshwater-farmed fish in the world. In this study, *C. idella* were exposed to different concentrations of 2-PE and MS-222. Using a combined transcriptomics and metabolomics analysis, the effects of these two anesthetics on the expression of *C. idella* genes and metabolic pathways were investigated, and then the mechanism of action of the two anesthetics on *C. idella* was analyzed to aid the process of selecting anesthetics in fish farming practice.

## 2 Materials and Methods

The use of experimental animals and the study protocol were reviewed and approved by the Ethics Committee of Shanghai Ocean University.

### 2.1 Fish Management andExperimental Design

Healthy fish (approximately 3 months old) with a body length of 16–18 cm and a weight of 27 ± 3 g (mean ± standard deviation) were obtained from an aquaculture farm located in Zhejiang, China. Fish were randomly divided into seven groups (control 1, control 2, residual detection group, 2-PE low-concentration group, 2-PE high-concentration group, MS-222 low-concentration group, and MS-222 high-concentration group) with 15 fish in each group, and each group was placed in a 512 L glass tank. For aquaculture, the water was aerated and exchanged 30 % daily, and water temperature was maintained at 22 ± 1°C. Consistent culture conditions were maintained for each group. Fish were fasted for 24 h before experimental procedures.

According to the pre-experimental results, relatively low concentrations of MS-222 (Carbon Dragon New Materials, Suzhou) and 2-PE (MACKLIN, Shanghai) were selected to achieve deep anesthetic effects in *C. idella* in 6 min. Fish were exposed to different concentrations of the anesthetics, and anesthesia time and status were recorded. The concentrations of the anesthetics used for the pre-experimental tests were as follows: MS-222 (0, 80, 150, and 200 mg/L) and 2-PE (0, 0.3, 0.6, and 1.2 mL/L). MS-222 was dissolved in distilled 10 mL water, whereas 2-PE was dissolved in 10 mL anhydrous ethanol, and then 10 L solutions of the anesthetics at various corresponding concentrations were prepared and kept in plastic barrels for further use. Fish in the pre-experimental group were quickly removed from the glass tanks using a net and released into plastic buckets containing different concentrations of the two anesthetics by. At a concentration of 80 mg/L for MS-222 and 0.6 mL/L for 2-PE, deep anesthesia was achieved in *C. idella* in 6 min, that is, the abdomen turned upward such that the fish stopped swimming but did not stop breathing and recovered in 5 min after being moved to water without anesthetic.

In this study, to explore the effects of different concentrations of anesthetics on fish, high- and low-concentration groups were established for each anesthetic: MS-222 low-concentration group (MD): 80 mg/L, MS-222 high-concentration group (MG): 200 mg/L, 2-PE low-concentration group (PD) and residual detection group (RD) : 0.6 mL/L, and 2-PE high-concentration group (PG): 1.2 mL/L.

### 2.2 Sampling

Seven groups were established in this study (MD, MG, PD, PG, RD, control 1 and control 2). In MD, MG, PD, PG and control 1 group 9 fish from 15 fish in each group were randomly selected for the whole experiment. Preparation method of anesthetic solution is consistent with that described for the pre-experiment. After 6 min of anesthesia, the brain tissue of fish was removed. In RD and control 2 group, 5 fish from 15 fish in each group were randomly selected for the whole experiment. Preparation method of anesthetic solution is consistent with that described for the pre-experiment. After 6 min of anesthesia, the brain tissue, muscle tissue and liver tissue of fish was removed.

For three randomly selected fish in MD, MG, PD, PG and control 1 group, after removing the brain tissue of the fish, the blood and dirt were quickly removed using precooled RNase-free water. The brain tissue was placed in a cryopreservation tube, labeled, and stored in liquid nitrogen to be used in the subsequent transcriptome analysis. The brain tissue samples of the remaining six fish in each group were removed, the residual blood was washed with normal saline, and the surface liquid was dried using filter paper. The brain tissue was placed in a cryopreservation tube, labeled, and stored in liquid nitrogen to be used in the subsequent metabolomics analysis.

For five randomly selected fish in RD and control 2 group, Their brain, muscle and liver tissues were frozen at -20°C for subsequent liquid chromatography residue detection.

### 2.3 Transcriptome Analysis

#### 2.3.1 RNA Extraction

Total RNA was isolated from each sample using TRIzol Reagent (Invitrogen Life Technologies), then, RNA concentration, quality, and integrity were determined using a NanoDrop spectrophotometer (Thermo Scientific) (Wu et al., 2016).

#### 2.3.2 Sequencing Library Preparation and Transcript Assembly

Both library preparation and RNA sequencing used 3 µg of RNA samples that passed quality standards. First, mRNA was purified from total RNA using poly-T oligonucleotide beads. Pyrolysis with divalent cations in Illumina specific lysis buffer at high temperature. Then, second-strand cDNA was synthesized using DNA polymerase I and RNase H, and the remaining drape was converted to a blunt end by exonuclease/polymerase activity, and the enzyme was removed. After the 3’ adenylation of DNA fragments, the Illumina PE aptamer oligonucleotides were ligated to the samples to prepare for the hybridization step (Hoseth et al., 2017; Ali et al., 2018). cDNA library fragments 400–500 bp in length were purified using the AMPure XP system (Beverly, California, USA). Illumina PCR Primer Cocktail was used to selectively enrich DNA fragments with linkers at both ends in 15 cycles of PCR. After purification (AMPure XP system), the product was quantified by Agilent high-sensitivity DNA analyzer on the bioanalyzer 2100 (Agilent). The sequencing library was then sequenced on NovaSeq 6000 platform (Illumina), (Liu et al., 2015).

#### 2.3.3 Transcriptome Sequencing

The samples were sequenced, and the raw data obtained in a FASTQ format were filtered with Cutadapt (v1.15) software to obtain clean data for further analysis. The filtered reads were aligned to the reference genome using TopHat2 upgraded HISAT2 software. Filtered reads were mapped to the reference genome using HISA T2 v2.0.5. HTSeq (0.9.1) was used to estimate the original expression level of the genes, and then the number of fragments per kilobase of transcript per million mapped reads (FPKM) was calculated to standardize the expression. Genes with |log2FoldChange| >1 and P value <0.5 identified using DESeq (1.30.0) were considered as differentially expressed genes (DEGs). Bidirectional clustering analysis of all genes was performed using the R package pheatmap (1.0.8).

#### 2.3.4 Gene Ontology and Kyoto Encyclopedia of Genes and Genomes Pathway Enrichment Analysis

All genes were mapped to the gene ontology (GO) database, and the number of DEGs enriched in each term was estimated. GO enrichment analysis of DEGs was performed using topGO (Wang et al., 2015); the significantly enriched GO terms of DEGs were identified, and the main biological functions of DEGs were determined. The Kyoto Encyclopedia of Genes and Genomes (KEGG) pathway analysis of DEGs was performed using ClusterProfiler (3.4.4) software.

### 2.4 Quantitative Reverse Transcription PCR Verification

To validate the RNA sequencing (RNA-Seq) results, four genes were randomly selected for a quantitative reverse transcription PCR (qRT-PCR) analysis using a 2× SYBR GREEN Master Mix (Vazyme Biotech Co., Ltd.). Primers were designed using the NCBI database, and β-actin was used as the reference gene. The thermal cycle of SYBR Green RT-PCR was as follows: 95°C for 10 min and 40 cycles at 95°C for 10 s and 60°C for 30 s. The genes to be validated are listed in **Table 1**, whereas the primers corresponding to the genes to be verified are listed in attachment.

**Table 1:** Genes to be validated by quantitative reverse transcription PCR.

Enzyme	Group	Homologous gene name	Homologous gene id	Gene id
SPLA2	MD/PD	cytosolic phospholipase A2 gamma-like isoform X1	107591457	CI01113194_00000034_00007377
	PD	group 3 secretory phospholipase A2-like	107712110	CI01000168_01110611_01115503
	MG	phospholipase B1, membrane-associated-like isoform X1	107721335	CI01000325_05510163_05515246
		HRAS-like suppressor 3	107756493	CI01002368_00000450_00001761
		cytosolic phospholipase A2 gamma-like isoform X1	107591457	CI01000426_00021251_00036674
	PG	hypothetical protein cypCar_00024211	KTG02580.1	CI01000170_00049973_00068447
ACSL	MG	long-chain-fatty-acid–CoA ligase ACSBG1-like	107757766	CI01000330_03692275_03699509
	MG/PG	long-chain-fatty-acid–CoA ligase 4-like	107732603	CI01000339_01860747_01871902
FASN	PG	fatty acid synthase	AGT29869.1	CI01000055_01852604_01874289

MD: MS-222 low-concentration group.

MG: MS-222 high-concentration group.

PD: 2-PE low-concentration group.

PG: 2-PE high-concentration group.

### 2.5 Metabolome Analysis

#### 2.5.1 Sample Extraction

The fish brain tissue samples were gradually thawed at 4°C. An appropriate amount of samples was added to the mixed solution of precooled methanol, acetonitrile and water (methanol: acetonitrile: water = 2:2:1, v/v). Samples were mixed by vortexing after low-temperature ultrasound for 30 min; then, they were allowed to stand at -20°C for 10 min, and then centrifuged at 14 000 rpm at 4°C for 20 min. The supernatant was vacuum-dried and redissolved in 100 μL acetonitrile aqueous solution (acetonitrile: water = 1:1, v/v), vortexed, centrifuged at 14000 rpm at 4°C for 15 min, and the supernatant was used as the sample for mass spectrometry.

#### 2.5.2 Liquid Chromatography/Mass Spectrometry Analysis

The analysis was performed using the Agilent 1290 Infinity LC Ultra-Performance Liquid Chromatography System (UHPLC) HILIC Column. The following conditions were used: column temperature 25°C; flow rate: 0.5 mL/min; injection volume 2 μL; mobile phase composition, A: water + 25 mM ammonium acetate + 25 mM ammonia, B: acetonitrile. The gradient elution procedure was as follows: 0–0.5 min, 95 % B; 0.5–7.0 min, B changed linearly from 95 % to 65 %; 7–8 min, B changed linearly from 65 % to 40 %; 8.0–9.0 min, B was maintained at 40 %; 9.0–9.1 min, B changed linearly from 40 % to 95 %; 9.1–12.0 min, B was maintained at 95 %. Samples were kept at 4°C in the automatic sampler during analysis.

After the samples were separated by HILIC chromatography, the primary and secondary spectra were collected using the AB Triple TOF 6600 mass spectrometer. ESI source conditions were as follows: ion source gas1 (Gas1): 60 psi, ion source gas2 (Gas2): 60 psi, curtain gas (CUR): 30 psi, source temperature: 600°C, IonSpray Voltage Floating: ± 5500 V (positive and negative modes), TOF MS scan m/z range: 60–1000 Da, product ion scan m/z range: 25–1000 Da, TOF MS scan accumulation time: 0.20 s/spectra, product ion scan accumulation time: 0.05 s/spectra; the secondary mass spectrum was obtained by information-dependent acquisition (IDA), and the high-sensitivity mode was adopted. Declustering potential (DP) was ±60 V (positive and negative modes), and collision energy was 35 ± 15 eV. IDA was set to exclude isotopes within 4 Da, and the number of candidate ions to monitor per cycle was 10. The final data were first subjected to metabolite structure identification and data preprocessing, then the quality of experimental data was evaluated, and finally data analysis was performed.

### 2.6 Analysis of 2-PE Residue in C. Idella by HPLC

#### 2.6.1 Sample Pretreatment

The quality of the sample was accurately weighed and recorded. The mass of muscle tissue was not more than 0.5000 g, and the sample tissue with good weight was transferred to the plastic centrifuge tube (50 mL). 10.0 mL acetonitrile-0.2 % acetic acid aqueous solution (V : V = 6 : 4) was added, ultrasonically extracted for 10 min, shaken for 15 min with vortex oscillator, centrifuged at 10000 r / min for 5 min, and the supernatant was taken with medical sterile disposable syringe (1 mL). The supernatant was purified by 0.22 μm organic needle-type filter, and bottled for detection.

#### 2.6.2 HPLC Parameter

Chromatographic column: ZORBAX SB-C18 (4.6×250mm, 5.0μm); mobile phase: acetonitrile - 0.2 % acetic acid aqueous solution (V : V = 6 : 4 ); flow rate : 0.3 mL / min; detection wavelength : 220 nm ; column temperature : 30°C ; sample volume: 3.0μL.

## 3 Results

### 3.1 Illumina Sequencing and Quality Assessment

To obtain the *C. idella* brain transcriptome expression profiles after treatment with MS-222 or 2-PE, 15 cDNA libraries were constructed. A total of 625 594 242 (128 251 100 in the control group, 127 897 444 in the MD group, 121 864 582 in the MG group, 129 677 354 in the PD group, and 117 894 762 in the PG group) raw reads were obtained. After quality control, 577 803 582 (118 058 270 in the control group, 118 156 658 in the MD group, 112 528 690 in the MG group, 119 818 882 in the PD group, 109 241 082 in the PG group) clean reads with a Q30% >92.97 % were obtained for subsequent analysis.

### 3.2 Analysis of Differentially Expressed Genes

The total mapping rate between reads and the reference genome was approximately 92 %. In total, 179 DEGs (74 upregulated and 105 downregulated) were identified between the control and MD groups; 887 DEGs (640 upregulated and 247 downregulated) were identified between the control and MG groups; 498 DEGs (238 upregulated and 260 downregulated) were identified between the control and PD groups; 514 DEGs (374 upregulated and 140 downregulated) were identified between the control and PG groups.

**Figure 1:**
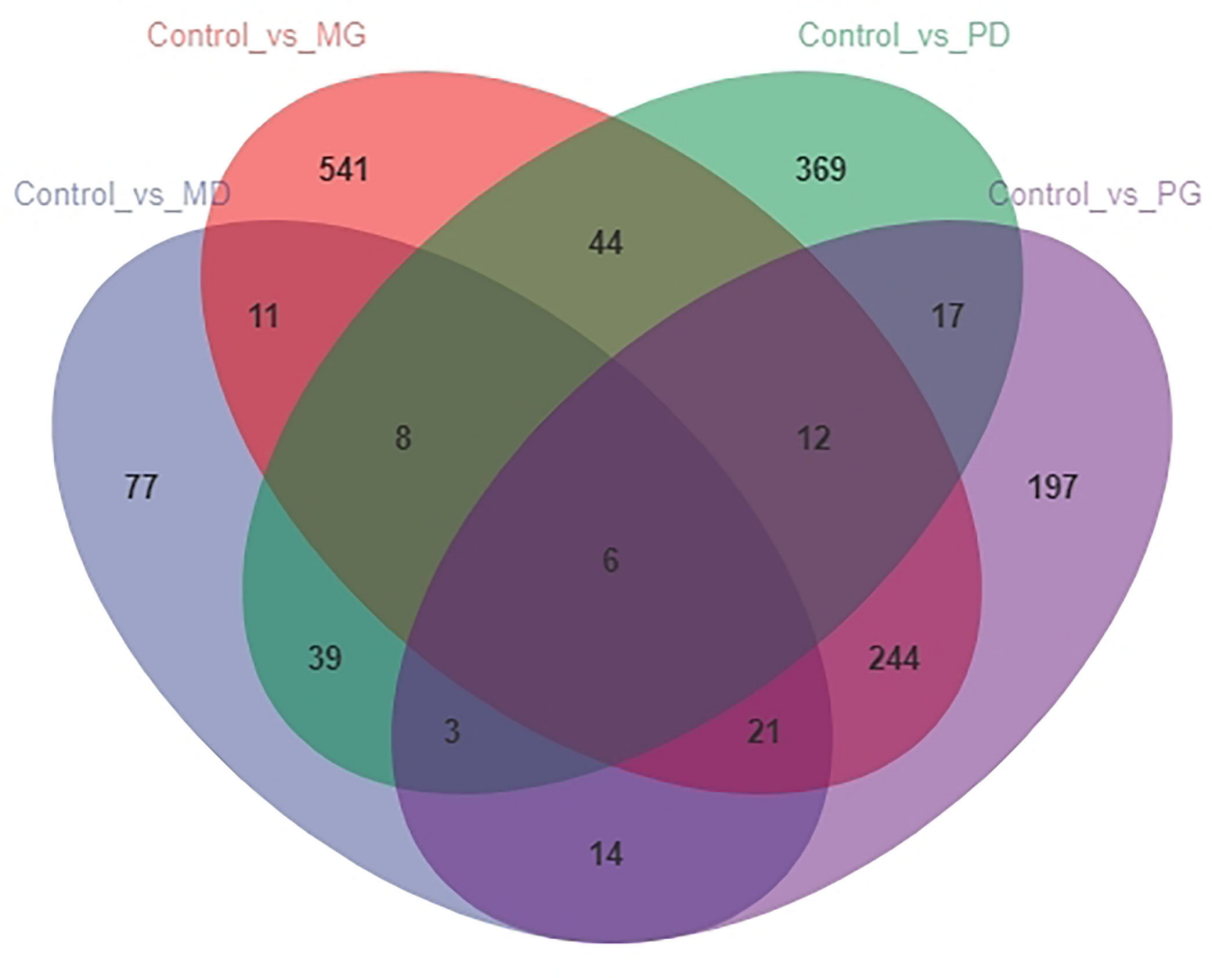
Venn diagram representing the number of differentially expressed genes among different treatment groups.

The analysis of the number of differentially expressed genes using a Venn diagram (**Figure 1**) showed that there were six common DEGs among all treatment groups. Four of them were encoded proteins; of the other two DEGs, one encodes immunoglobulin Z heavy chain, and the other encodes the protein shisa-3 homolog isoform X1. Moreover, there were many common DEGs between different treatment groups.

#### 3.2.1 Gene Ontology Annotation of Differentially Expressed Genes

**Figure 2:**
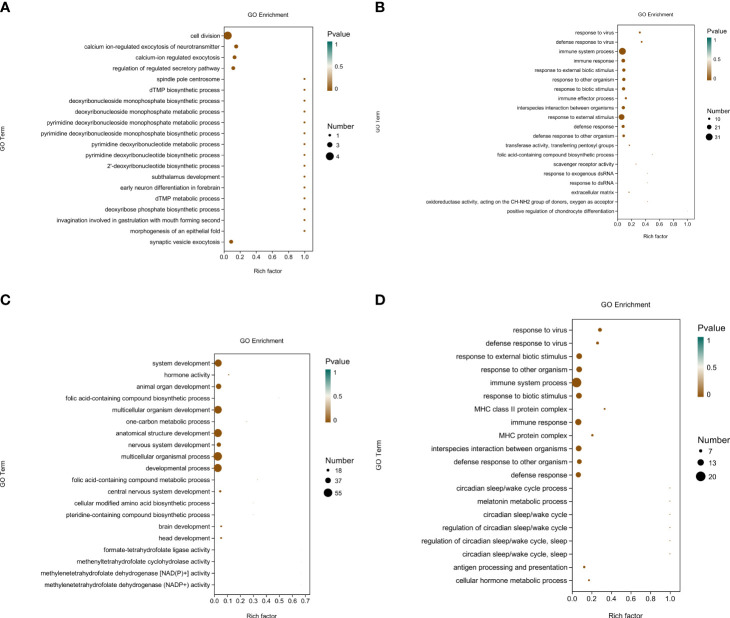
The top 20 GO terms in each group. **(A)** Gene ontology (GO) annotation of the top 20 differentially expressed genes in the brain of *Ctenopharyngodon idella* following treatment with MS-222 at a low concentration. **(B)** Gene ontology (GO) annotation of the top 20 differentially expressed genes in the brain of *Ctenopharyngodon idella* following treatment with MS-222 at a high concentration. **(C)** Gene ontology (GO) annotation of the top 20 differentially expressed genes in the brain of *Ctenopharyngodon idella* following treatment with 2-phenoxyethanol (2-PE) at a low concentration. **(D)** Gene ontology (GO) annotation of the top 20 differentially expressed genes in the brain of *Ctenopharyngodon idella* following treatment with 2-phenoxyethanol (2-PE) at a high concentration.The abscissa indicates the richness factor (the number of DEGs annotated to a GO term/the total number of genes annotated to a GO term), and the ordinate indicates the GO terms. The size of the circles in the figure represents the number of genes annotated to the corresponding GO term (upregulated or downregulated in relation to the gene set selected in the analysis). The depth of color represents the level of dominance.

After completing the differential gene analysis, the DEGs were annotated using the GO database. According to the genome annotation data, the DEGs between groups were classified to analyze the functions of these genes. **Figure 2** show the top 20 GO terms in each group; most are related to Biological Processes.

#### 3.2.2 Kyoto Encyclopedia of Genes and Genomes Pathway Analysis of Differentially Expressed Genes

In this study, 329 DEGs identified between the control group and MD group were associated with 152 known KEGG pathways, of which 29 KEGG pathways were significantly enriched, and 14 pathways were extremely significantly enriched; 1550 DEGs identified between the control group and MG group were associated with 273 known KEGG pathways, of which 40 KEGG pathways were significantly enriched, and 27 pathways were extremely significantly enriched; 806 DEGs identified between the control group and PD group were associated with 234 known KEGG pathways, of which 25 KEGG pathways were significantly enriched, and 17 pathways were extremely significantly enriched; 1079 DEGs identified between the control group and PG group were associated with 239 known KEGG pathways, of which 49 KEGG pathways were significantly enriched, and 41 pathways were extremely significantly enriched. The KEGG pathways enriched with DEGs in each comparison were mostly related to immunity and metabolism. There were no significant differences in key genes related to cortisol production and metabolism among the treatment groups compared with the control group. The significant enrichment pathways of differentially differentiated genes in each treatment group are shown in the attachment.

### 3.3 Analysis of Differential Metabolites

In total, 304 metabolites were identified by using a combination of positive and negative ion modes in mass spectrometry; the number of metabolites identified using the positive and negative ion mode was 169 and 135, respectively. The proportion of metabolites according to their chemical taxonomy is shown in the attachment.

**Figure 3:**
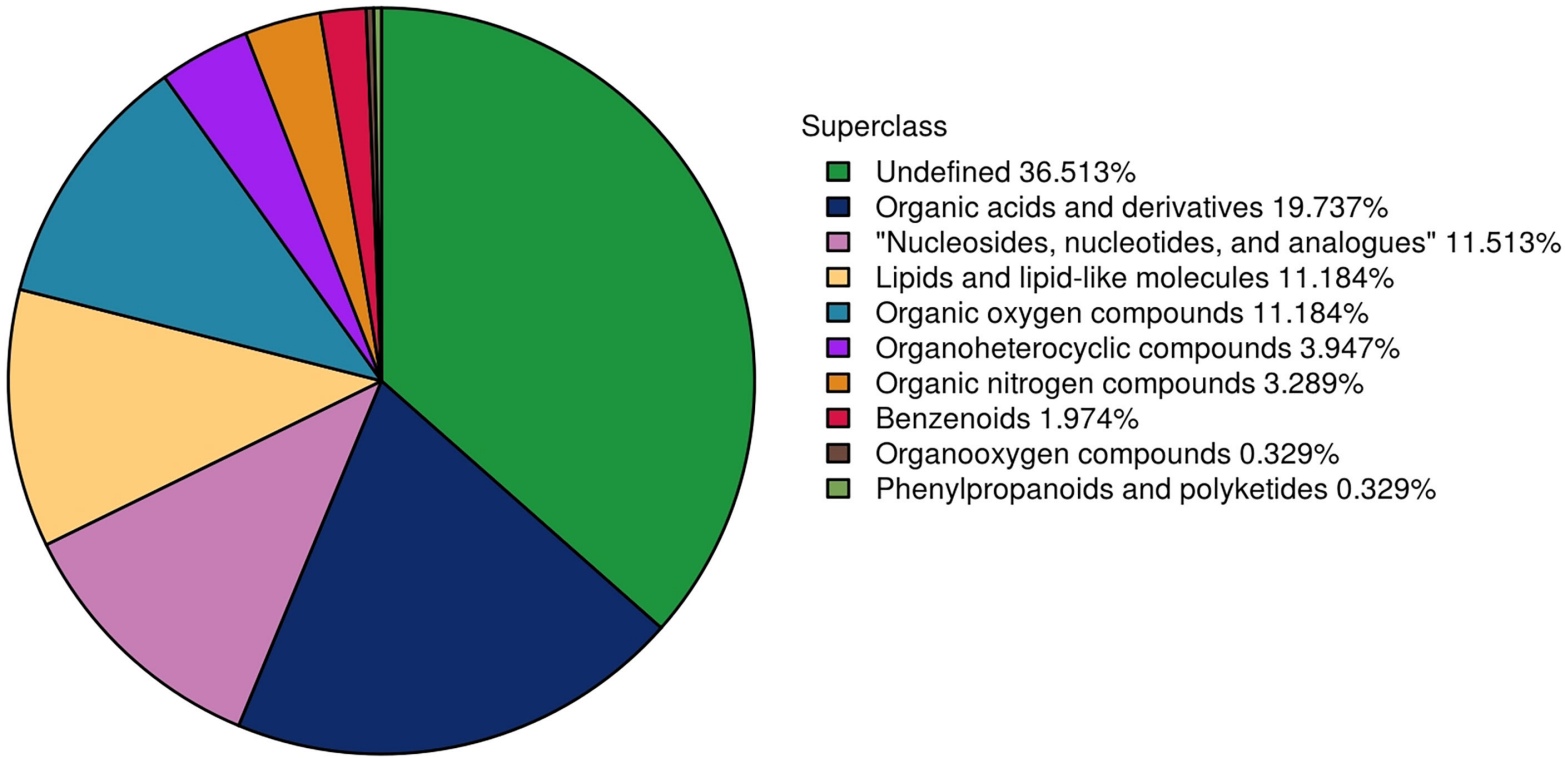
The differential metabolites detected by metabolomics between the treatment group and the control group were classified according to their chemical structure.

In the MD group, 27 differential metabolites were identified, most notably enriched in Arachidonic acid metabolism, PPAR signaling pathway, and ABC transporters. In the MG group, 36 differential metabolites were identified, most notably enriched in Arachidonic acid metabolism, PPAR signaling pathway, and Carbohydrate digestion and absorption. In the PD group, 15 differential metabolites were identified, most notably enriched in Arachidonic acid metabolism, Lysosome, and ABC transporters. In the PG group, 14 differential metabolites were identified, most notably enriched in Tyrosine metabolism; cAMP signaling pathway; and Alanine, aspartate, and glutamate metabolism. The main metabolic pathways enriched with the differential metabolites of treatment groups except the PG group were related to arachidonic acid metabolism. The main metabolites involved in these pathways include linoleic acid and some cytochrome P450 (CYP)-catalyzed arachidonic acid products, such as 12(R)-HETE and 20-HETE. But there was no significant difference in cortisol expression between the treatment groups and the control group. Differential metabolites in each group are listed in attachment. **Figure 3** shows proportion of metabolites according to their chemical taxonomy.

### 3.4 Combined Transcriptomics/Metabolomics Analysis

On the basis of the results of the metabolomics and transcriptomics analysis, the differentially expressed metabolites and transcripts were identified; then, the transcripts of related enzymes were identified on the basis of the metabolites in the KEGG database; then, metabolites and related transcripts were mapped to related metabolic pathways.

A common pathway associated with differentially expressed metabolites and transcripts in the MD, MG, and PD groups (p <0.05) was Linoleic acid metabolism (map00591). In the MG group, other seven major metabolic pathways were Glycerophospholipid metabolism (map00564), Arachidonic acid metabolism (map00590), alpha-Linolenic acid metabolism (map00592), Fatty acid degradation (map00071), Fatty acid biosynthesis (map00061), Biosynthesis of unsaturated fatty acids (map01040), and Choline metabolism in cancer (map05231). Five major metabolic pathways were identified in PG group: Purine metabolism (map00230), Sphingolipid signaling pathway (map04071), Vascular smooth muscle contraction (map04270), cAMP signaling pathway (map04024), and Prion disease (map05020).

**Figure 4:**
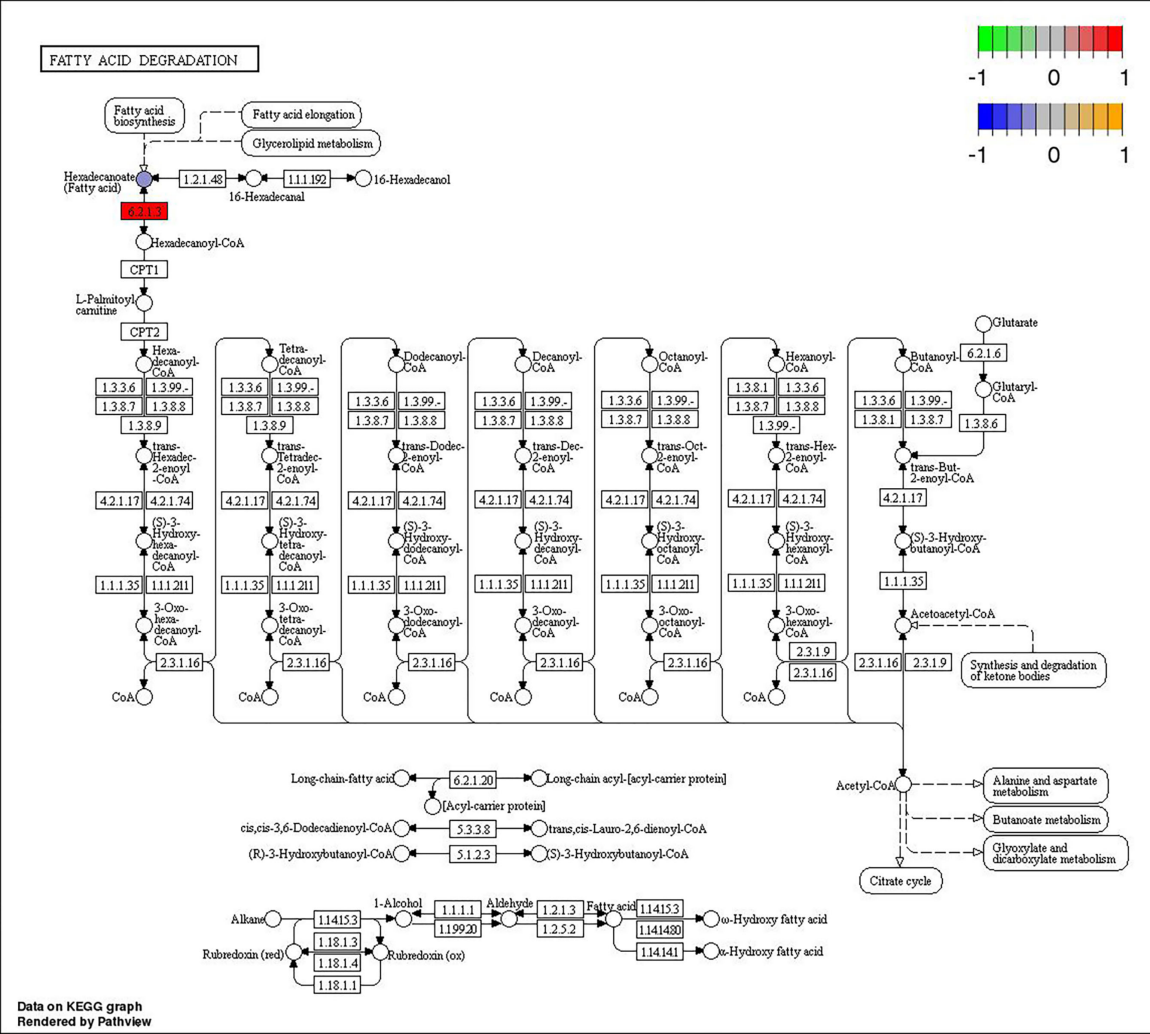
Changes in the fatty acid degradation pathway in *Ctenopharyngodon idella* brain after MS-222 high-concentration treatment.

**Figure 5:**
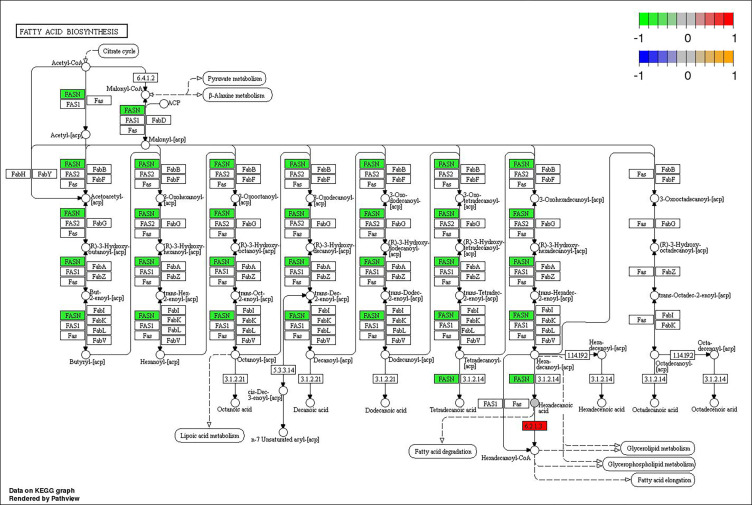
Changes in the fatty acid biosynthesis pathway in *Ctenopharyngodon idella* brain after 2-phenoxyethanol (2-PE) high-concentration treatment.

**Figure 6:**
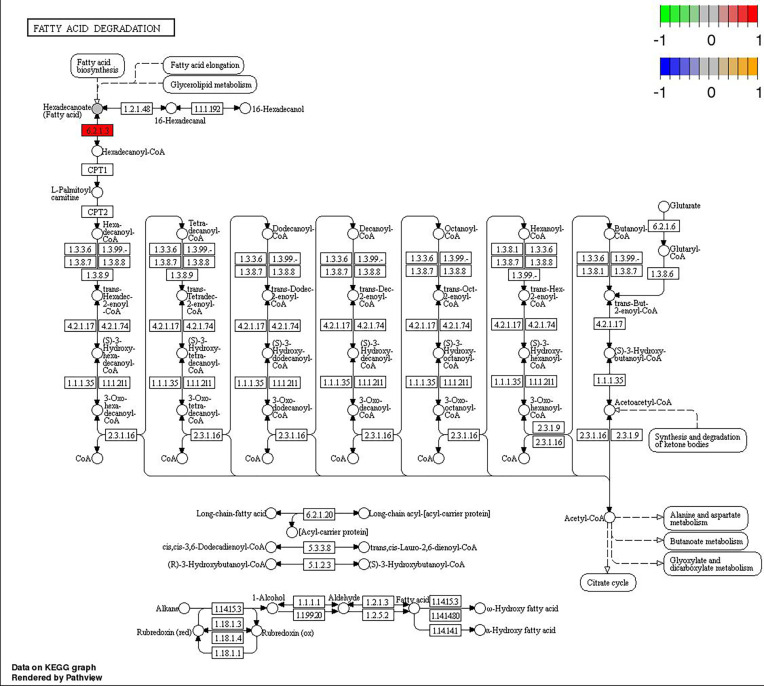
Changes in the fatty acid degradation pathway in *Ctenopharyngodon idella* brain after 2-phenoxyethanol (2-PE) high-concentration treatment.

Through joint analysis, it was found that the expression of secretory phospholipase A2 (*SPLA2*) was significantly different in each treatment group; it was significantly downregulated in the MD, PD, and PG groups and significantly upregulated in the MG group. Moreover, joint analysis showed that in the MG group, the upregulation of long-chain acyl-CoA synthetase (*ACSL*) downregulated hexadecanoic acid (map00071, **Figure 4**). However, in the PG group, although *ACSL* was upregulated, the downregulation of hexadecanoic acid was reduced owing to the significant downregulation of fatty acid synthase animal type (*FASN*; **Figures 5**, **6**).

Therefore, on the basis of transcriptome results, qRT-PCR was used to verify the DEGs regulating *SPLA2* and *FASN*.

### 3.5 qRT-PCR Analysis

Through joint analysis, it was found that the genes that controlled the expression of *SPLA2* in *C. idella* differed in different treatment groups. Although these genes correspond to the same homologous genes in the KEGG database, they are different genes in the *C. idella* genome, with different base sequences.

**Figure 7:**
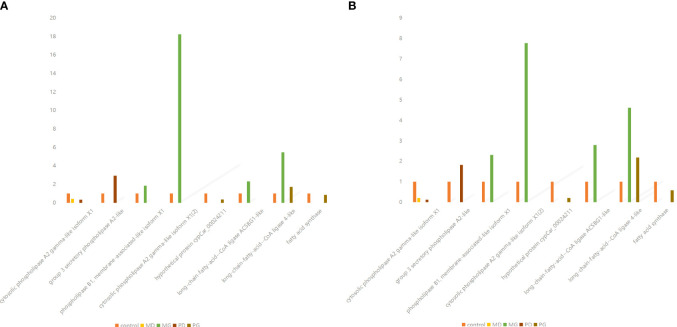
Comparison of qpcr verification results with transcriptome analysis results. **(A)** Quantitative reverse transcription PCR (qRT-PCR) verified the relative expression of differentially expressed genes in *Ctenopharyngodon Idella* brain. Gene names are presented on the abscissa, and fold change values are presented on the ordinate. **(B)** Relative expression of differentially expressed genes (DEGs) in in *Ctenopharyngodon idella* brain obtained by RNA-Seq. Gene names are presented on the abscissa, and fold change values are presented on the ordinate.

After verification, it was found that the results were consistent with the transcriptomics results, confirming the conclusions of the transcriptomics analysis (**Figure 7**).

### 3.6 Determination of 2-PE Residue by HPLC

The results showed that 2-PE had a good linear relationship with peak area in the concentration range of 5– 300 mg / L under the chromatographic parameters 300 mg / L.

After the grass carp were bathed in 0.6 mg/L 2-PE solution for 6 min, the residue of 2-phenoxyethanol in brain, muscle and liver of grass carp were 0.203mg/kg, 0.063mg/kg and 0.103mg/kg, respectively.

## 4 Discussion

Studies have indicated that stress response, which negatively affects fish, can be alleviated by anesthesia (Le et al., 2019). However, there are many kinds of anesthetics, and the effects of various anesthetics on the physiological metabolism of different fish need to be further studied. The present study combined transcriptomics and metabolomics to investigate the toxic effects of 2-PE and MS-222 on *C. idella* and their effects on physiological metabolism, the unknown mechanism of action of related anesthetics in *C. idella* was supplemented and discussed.

MS-222, a sodium channel blocker, is the most commonly used anesthetic in aquatic organisms, including fish (Lidster et al., 2017). Its main mechanism of action is similar to that of other anesthetics; it inhibits sodium entry into excitable brain cells, thereby blocking action potential (Butterworth and Strichartz, 1990). In 1985, Lewis et al. (1985) found that MS-222 had adverse effects on the olfactory organs of channel catfish; they reported that the drug destroyed the cilia of the olfactory sensory epithelium. However, in 1988, Quinn et al., 1988) did not report a negative effect of MS-222 on the chemical sensory ability of Pacific salmon through experiments. In 2017, (Nordgreen et al., 2014) reported that MS-222 had no effect on several commonly used behavioral parameters of zebrafish. However, in 2018, Felix et al. (2018) showed that MS-222 was associated with a risk of teratogenic mortality in early zebrafish embryos.

In 2005, Marsic-Lucic et al. (2005) showed that 2-PE was safer than propiscin as an anesthetic in young sea bass (*Dicentrarchus labrax* L.). Velisek et al. (2007) showed that the use of 2-PE at a concentration of 0.30 mL/L did not cause any irreversible damage to sheatfish. (Vrskova and Modra (2012) reported in 2012 that 2-PE at concentrations higher than 300 mg/L induced embryonic growth inhibition in frogs and posed a teratogenic risk. A 2014 study by (Mitjana et al., 2014; Bonastre et al. 2014) noted that repeated exposure to 2-PE resulted in some tolerance of juvenile angel fish to sedation and anesthetics, indicating that angel fish produce compensatory effects by reducing the number of GABA receptors, thereby prolonging the sedation and anesthesia time. In 2016, Akbary et al. (2016) reported that 2-PE can be used as an anesthetic at a concentration of 0.9 mL/L, which has certain safety for bighead carp. Rapid anesthesia at this concentration has the least effect on physiological stress, and 2-PE cannot be regarded as a dangerous substance at this concentration.

This further indicates that anesthetics have different toxicity in different species or in different growth stages of the same species. Therefore, *C. idella* was used as a representative species to further understand the effects of these two anesthetics on aquatic animals at the transcriptomic and metabolomic level.

### 4.1 Effects on Differential Gene Expression

In the transcriptomics analysis, results showed that that when the dosage of the two anesthetics achieved roughly the same depth of anesthesia in 6 min, the number of DEGs in the MD group compared with the control was 179, whereas the number of DEGs in the PD group compared with the control was 498. However, when the drug concentration was increased, there were 887 DEGs in the MG group and 514 DEGs in the PG group compared with the control. The DEGs associated with MS-222 were enriched in immune pathways, lipid metabolism, amino acid metabolism and various signaling pathways, whereas the DEGs associated with 2-PE were enriched in immunity and amino acid metabolism.

### 4.2 MS-222 Reduced the Expression of 20-HETE

The liquid chromatography/mass spectrometry showed that in the MS-222 treatment groups, the metabolites with the most significant differences between the two concentration groups under negative ion detection were 12(R)-HETE and 20-HETE, and those with the most significant differences between the two concentration groups under positive ion detection were 4-aminobenzoate and 20-HETE. Thus, MS-222 anesthesia had the greatest impact on the formation and metabolism of 20-HETE in *C. idella*. 20-HETE is an active substance catalyzed by CYP4A/4F to produce arachidonic acid (Garcia and Schwartzman, 2017). 20-HETE induces oxidative stress by promoting eNOS uncoupling, interferes with endothelial cell function, and increases blood pressure (Tunctan et al., 2010). The expression of 20-HETE in each sample was significantly downregulated in the MS-222 treatment groups. This may be because MS-222 reduces 20-HETE levels, thereby alleviating oxidative stress in fish and helping induce complete anesthesia.

### 4.3 Upregulation of Salidroside and Linoleic Acid Expression in Response to 2- Phenoxyethanol Toxicity

Salidroside was the most significant metabolite detected in the negative ion mode in the 2-PE high- and low-concentration treatment groups. The main effects of salidroside include anti-hypoxic, anti-inflammatory, anti-viral, anti-cancer, anti-fatigue, immune-boosting, hepatoprotective and neuroprotective effects (Wang et al., 2018). Zhao, XY et al. found that salidroside inhibited the activation of caspase-3/9 and cleavage of poly(ADP-ribose) polymerase induced by endogenous H_2_O_2_. It also decreased the expression of Bax and rescued the balance of pro- and anti-apoptotic proteins (Zhao et al., 2013). Guan et al. found that the antioxidant effects of salidroside were associated with downregulation of free cytosolic Ca^2+^ and ROS scavenging *via* a cAMP-dependent pathway (Guan et al., 2011). The fold change value of salidroside between 2-PE high- and low-concentration groups was ≥50, it indicates that the drug has a great influence on the expression of this metabolite, and 2-PE may affect the upstream and downstream pathways of this metabolite.

In the low-concentration group of 2-PE, linoleic acid was significantly upregulated, whereas in the high-concentration group of 2-PE, linoleic acid was upregulated but not significantly upregulated. Linoleic acid is a fundamental member of ω-3 unsaturated fatty acids, which can be transformed into γ-linolenic acid and then into arachidonic acid (Xu, 2013). Min, (2012) reported that linoleic acid and methyl linoleate reduce the level of inflammation-related factors and have a significant improvement in acute and chronic inflammation. Wang etal. (2009) confirmed that linoleic acid increases mitogen-induced lymphocyte proliferation, enhances lymphocyte activity, enhances macrophage phagocytosis, increases cell-mediated immunity, influences immunoglobulin and antibody production, and directly affects the immune activity.

These findings suggest that 2-PE is more likely to be toxic to *C. idella*, in response to which fish begin to secrete anti-inflammatory substances. The fact that the same results were observed in the high-concentration and low-concentration 2-PE groups also indicates high confidence.

Other significant differential metabolites from the two drug treatment groups were also inconsistent, for example, in mS-222 treatment group, l-gulonic gamma-lactone, 4-aminobenzoate, and 3. Alpha. -mannobiose were also important differential metabolites. D-Aspartic acid and other important metabolites were found in 2-PE treatment group, indicating that the anesthetic mechanism of 2-PE in fish was different from that of MS-222.

### 4.4 No Significant Difference in Cortisol Levels Among Treatment Groups

Cortisol is one of the hormones involved in stress response. After stress, serum cortisol concentration increases (Bowser, 1985; Semenkova et al., 2010). Transcriptome results showed that genes related to cortisol secretion were differentially expressed between groups; for example, *UCN2–3* was significantly differentially expressed between MD and PD, whereas *SGK1* was significantly differentially expressed among MG, PD, and PG. However, there were no significant differences in the corticotropin-releasing hormone gene (*CRH*) and its receptors *CRHR1* and *CRHR2*. There was also no significant difference in the salt corticosteroid receptor gene *MR3C1* and *NR3C2* between the treatment groups and the control group. Metabolic group results showed no significant difference in cortisol levels between the treatment groups and the control group. Therefore, the two anesthetics had no significant effect on the cortisol level of *C. idella* under the experimental conditions. This is also consistent with the findings of Jerez-Cepa et al. (2019); when they explored the effect of MS-222 on the hypothalamic–pituitary–interrenal axis of *Sparus macrocephalus*, they found no significant difference in the expression of plasma cortisol in fish treated with MS-222.

### 4.5 Effects of Two Anesthetics on Arachidonic Acid Metabolism and its Upstream and Downstream Metabolic Pathways

Joint analysis showed that the expression of secretory phospholipase A2 (SPLA2) was significantly different in each treatment group. SPLA2 is an upstream regulator of the eicosane cascade that provides free fatty acids to cyclooxygenase (COX), lipoxygenase, and cytochrome P450 acid, resulting in various inflammatory mediators (Nikolaou et al., 2019). They were significantly downregulated in the MD, PD, and PG groups and significantly upregulated in the MG group. Moreover, in the MD and PD groups, the significantly different metabolites mapped to the same metabolic pathway as SPLA2 happened to be linoleic acid that can be converted to arachidonic acid. In the MG group, phosphatidylcholine is mapped to the same metabolic pathway with SPLA2; phosphatidylcholine is hydrolyzed by SPLA2 to produce linoleic acid. Therefore, MS-222 treatment affected the arachidonic acid metabolic pathway of *C. idella*; low concentrations of 2-PE affected the arachidonic acid metabolic pathway by affecting the linoleic acid metabolic pathway in *C. idella*.

In the MD group, SPLA2 was significantly downregulated, and linoleic acid was significantly upregulated. This may be due to the negative impact of MS-222 on arachidonic acid metabolic pathway, which is also consistent with the significant downregulation of arachidonic acid metabolites 20-HETE and 12(R)-HETE in the MD group.

In the MG group, although SPLA2 was significantly upregulated, and phosphatidylcholine was also significantly upregulated, 20-HETE and 12(R)-HETE remained significantly downregulated, which further indicated that MS-222 inhibited arachidonic acid metabolism in *C. idella*. This change in phosphatidylcholine may be related to toxicity-induced oxidative stress, reactive oxygen species (ROS) production, and lipid peroxidation and may change the stability of the cell membrane. This may be considered one of the toxic effects of anesthetics on *C. idella*.

However, in the PD group, although the expression of SPLA2 was downregulated and the expression of linoleic acid was upregulated, the arachidonic acid metabolites 20-HETE and 12(R)-HETE were significantly upregulated. This may be due to a positive effect of 2-PE on arachidonic acid metabolism, which makes the feedback regulation of metabolites on the SPLA2. However, this finding needs to be further validated.

Unlike the other three groups, the PG group showed a significant downregulation of SPLA2, and the expression of 12(R)-HETE and 20-HETE was slightly but not significantly increased, indicating that the 2-PE on may promote arachidonic acid metabolism at a low concentration and inhibit it at a high concentration.

Furthermore, the joint analysis showed that, unlike in the MG group, although ACSL was still upregulated in the PG group, the downregulation of hexadecanoic acid was reduced due to the significant downregulation of FASN. Whether ACSL and FASN have an antagonistic relationship remains to be studied.

These findings confirm that MS-222 and 2-PE affect arachidonic acid metabolism or its upstream pathways such as linoleic acid metabolism and phosphatidylcholine metabolism in *C. idella*; however, it remains to be studied whether other anesthetics have the same effect in *C. Idella* and whether the two anesthetics have the same effect on other fish.

## 5 Conclusions

The metabolism of arachidonic acid in C. idella was inhibited by MS-222. 2-PE affected the upstream and downstream metabolic pathways of arachidonic acid metabolism, thereby affecting the metabolism of arachidonic acid. Both anesthetics inducedsedation by affecting related metabolites that affected the stress response and autoimmunity. However, rapid use of anesthetics causes more than ten-fold change in the secretion of relatedmetabolites in C. idella in the short term, and whether it causes metabolic disorders and progressive toxicity in C. idella remains to be studied. Much research remains to be done on themechanisms of anesthetics, including the effects of different environmental conditions on anesthetics and the effects of anesthetics on fish of different ages.

## Data Availability Statement

The datasets presented in this study can be found in online repositories. The names of the repository/repositories and accession number(s) can be found in the article/**Supplementary Material**.

## Ethics Statement

The animal study was reviewed and approved by Ethics Committee of Shanghai Ocean University.

## Author Contributions

WT was responsible for experimental design, experimental operation, data analysis and paper writing. WY is responsible for data query and experimental operation. LX is responsible for data enquiries. GX is responsible for manuscript revision. HK is responsible for experimental design, data analysis and financial support. All authors contributed to the article and approved the submitted version.

## Funding

This study was supported by the National Key R&D Program of China(2019YFD0900102), China, the Science and Technology Commission of Shanghai Municipality [grant number 18391901500], and the NationalNatural Resources Platform and Shanghai Ocean University KnowledgeService Platform, China. Other authors provided material support for this paper and assisted in data analysis.

## Conflict of Interest

The authors declare that the research was conducted in the absence of any commercial or financial relationships that could be construed as a potential conflict of interest.

## Publisher’s Note

All claims expressed in this article are solely those of the authors and do not necessarily represent those of their affiliated organizations, or those of the publisher, the editors and the reviewers. Any product that may be evaluated in this article, or claim that may be made by its manufacturer, is not guaranteed or endorsed by the publisher.
