# Supplementary material for: Combined Transcriptomics and Metabolomics Analyses in Grass Carp Under Anesthetic Stress
Source: Front Cell Infect Microbiol. 2022 Jul 11;12:931696. doi: 10.3389/fcimb.2022.931696 (PMC9309352; doi:10.3389/fcimb.2022.931696)
Supplement: Supplementary file 1 [file DataSheet_1.docx]

The enriched pathways of each group.


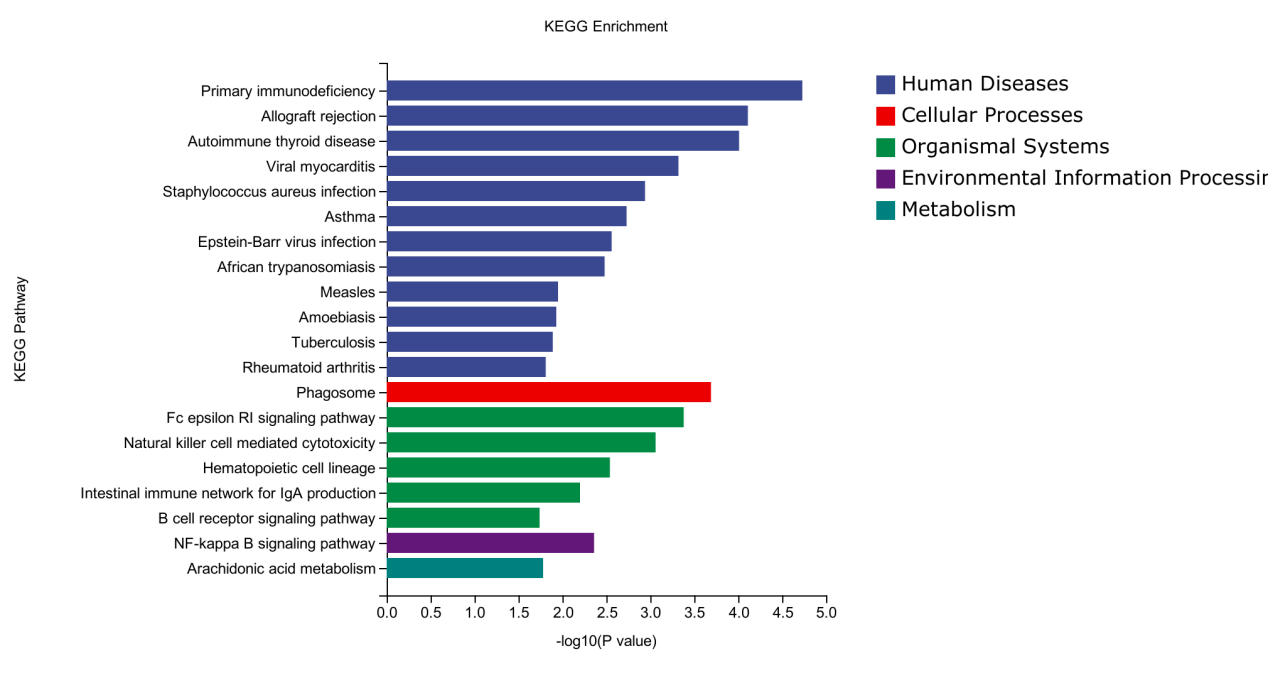


Fig. 1. Histogram of KEGG pathways enriched in differentially expressed genes in *Ctenopharyngodon idella* brain after treatment with MS-222 at a low concentration.


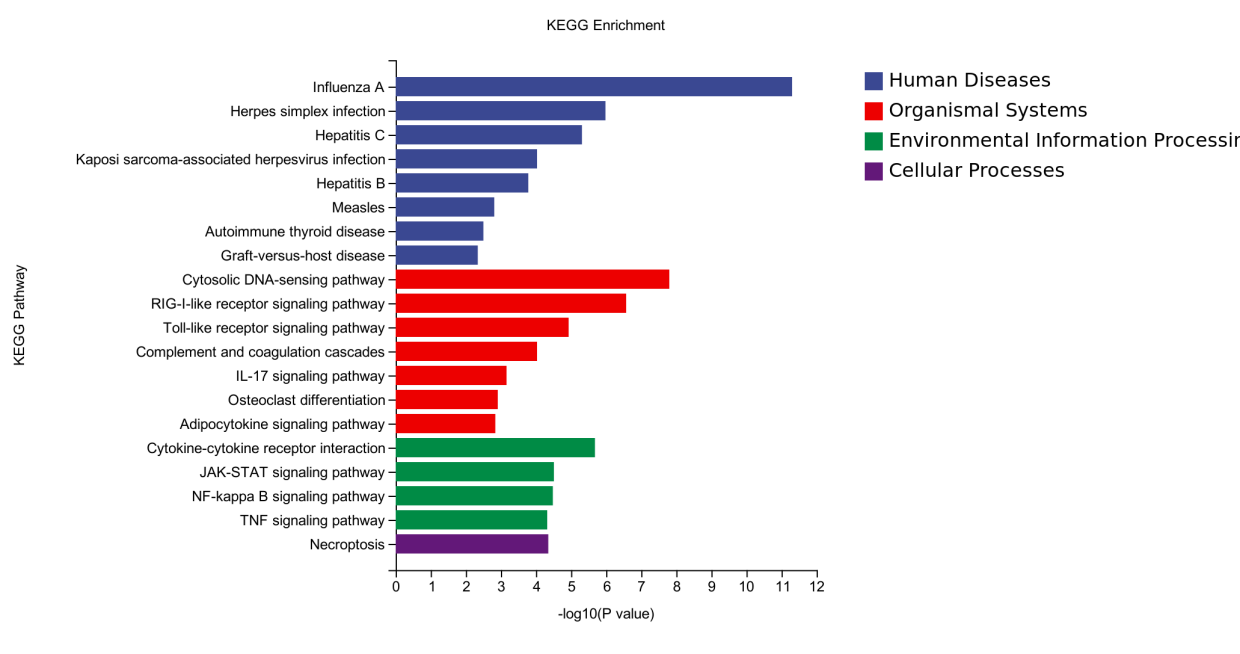


Fig. 2. Histogram of KEGG pathways enriched in differentially expressed genes in *Ctenopharyngodon idella* brain after MS-222 high-concentration treatment.


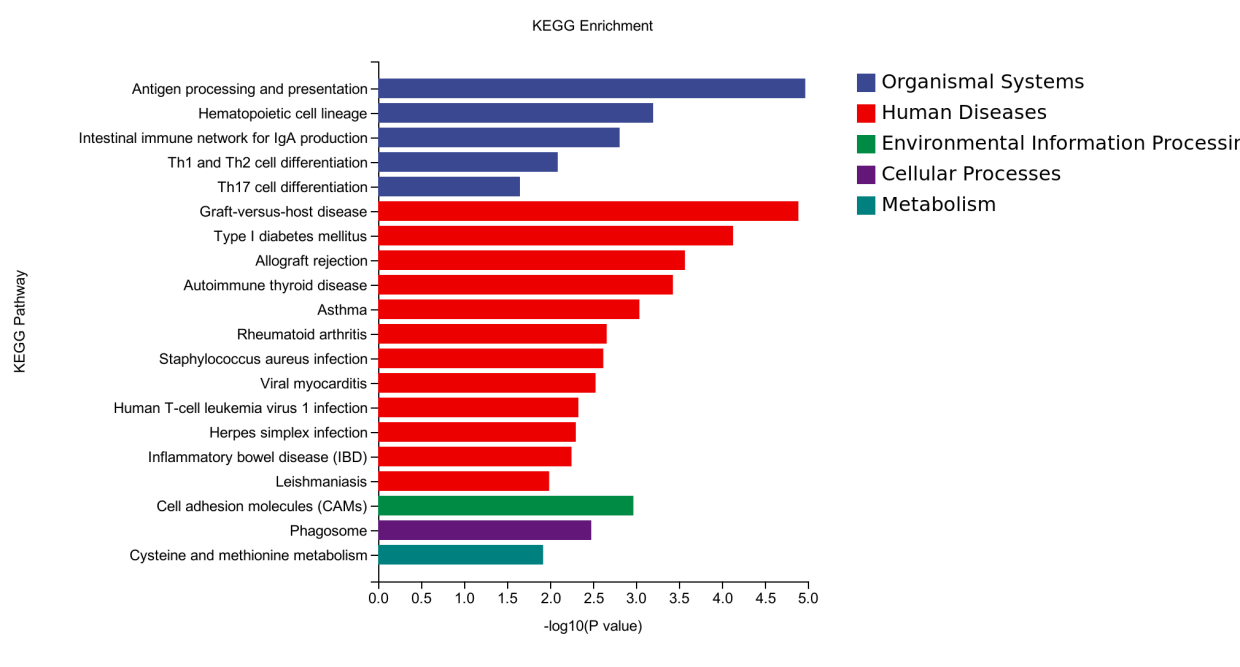


Fig. 3. Histogram of KEGG pathways enriched in differentially expressed genes in *Ctenopharyngodon idella* brain after 2-phenoxyethanol (2-PE) low-concentration treatment.


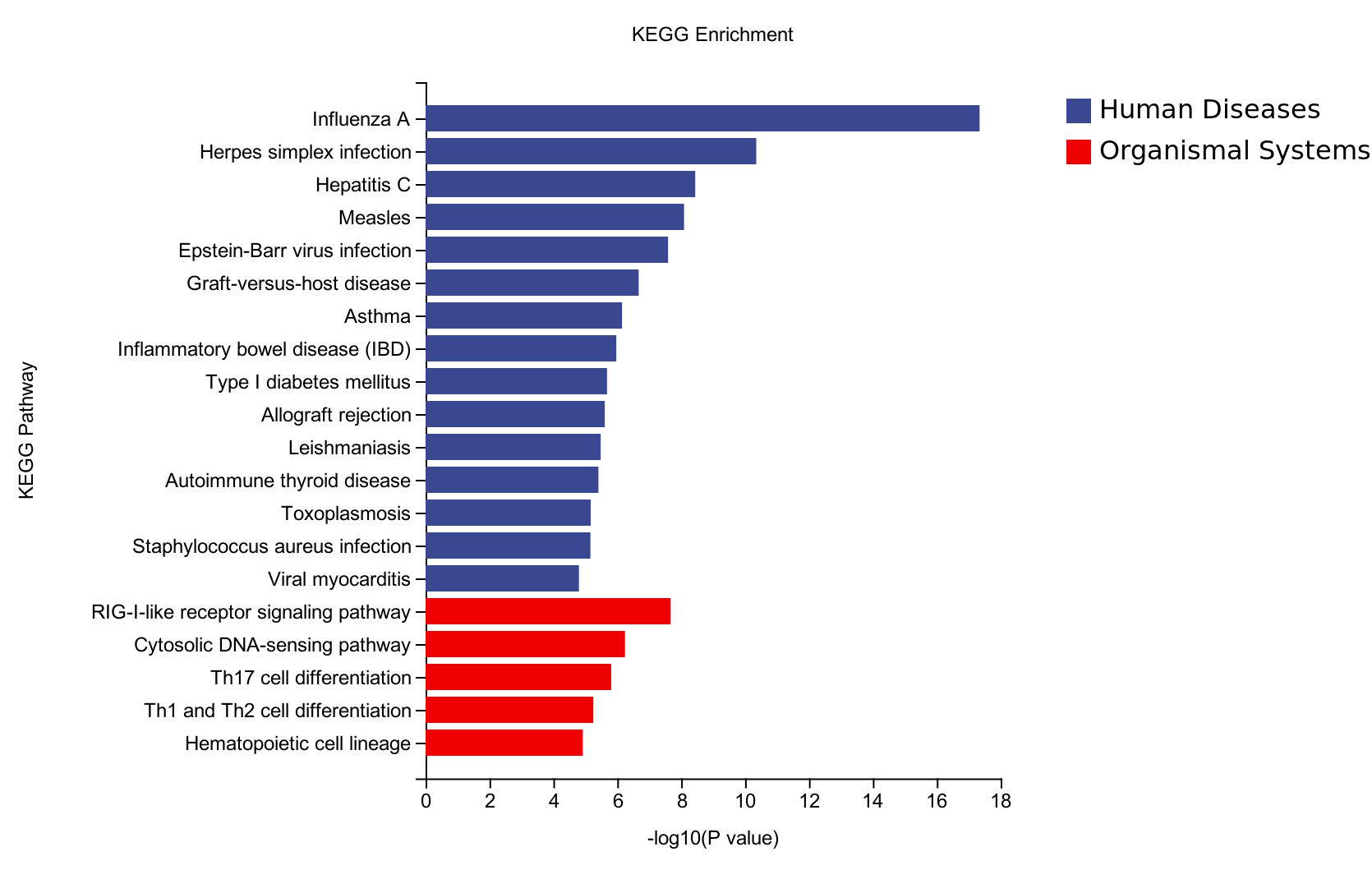


Fig. 4. Histogram of KEGG pathways enriched in differentially expressed genes in *Ctenopharyngodon idella* brain after 2-phenoxyethanol (2-PE) high-concentration treatment.
