# Supplementary material for: Combined Transcriptomics and Metabolomics Analyses in Grass Carp Under Anesthetic Stress
Source: Front Cell Infect Microbiol. 2022 Jul 11;12:931696. doi: 10.3389/fcimb.2022.931696 (PMC9309352; doi:10.3389/fcimb.2022.931696)
Supplement: Supplementary file 2 [file DataSheet_2.docx]

Table 1. Significant differential metabolites in each treatment group.

| Group | Name | VIP | Fold change | p-value |
| --- | --- | --- | --- | --- |
| MD | 4-Aminobenzoate | 4.771569372 | 57.34229189 | 1.06971E-07 |
|  | 12(R)-HETE | 21.5309454 | 0.085741221 | 1.34278E-06 |
|  | 20-Hydroxyarachidonic acid | 5.770630571 | 0.083475025 | 1.9107E-05 |
|  | 20-HETE | 1.801758371 | 0.029240426 | 3.93778E-05 |
|  | 5(S)-HETE | 13.12066806 | 0.063036475 | 0.000851949 |
|  | 12-oxo-ETE | 1.388340515 | 0.173600007 | 0.000988328 |
|  | L-Gulonic gamma-lactone | 1.27268556 | 2.626570717 | 0.001745799 |
|  | 8(S)-HETE | 1.58626862 | 0.167448388 | 0.002380937 |
|  | 13(S)-HODE | 1.405523016 | 1.684767181 | 0.002720697 |
|  | D-Mannose | 4.284639505 | 2.781403682 | 0.002762864 |
|  | Adenine | 1.358484377 | 0.557207387 | 0.003737201 |
|  | Thr-Ala | 1.018498545 | 2.422537168 | 0.003855891 |
|  | Prostaglandin E2 | 1.203623416 | 0.601387519 | 0.007200189 |
|  | LTB4 | 3.15243653 | 0.11153342 | 0.007810638 |
|  | alpha-Tocopherol (Vitamin E) | 1.193321595 | 0.347532823 | 0.00814559 |
|  | Linoleic acid | 1.412467701 | 2.228598067 | 0.008449849 |
|  | His-Ile | 1.073543477 | 2.20664151 | 0.014933493 |
|  | Adenosine | 3.445397555 | 0.378764322 | 0.015315662 |
|  | L-Proline | 1.067789914 | 1.504330315 | 0.018390402 |
|  | 5(S)-HpETE | 2.139715086 | 0.205736325 | 0.018979297 |
|  | Isomaltose | 2.499363509 | 3.393569982 | 0.024989035 |
|  | 3.alpha.-Mannobiose | 2.562513718 | 3.35316292 | 0.025017798 |
|  | L-Leucine | 1.274939106 | 1.338337804 | 0.027093034 |
|  | Maltotriose | 1.808331286 | 5.483108255 | 0.034161611 |
|  | Histamine | 1.283173267 | 0.795811296 | 0.034378809 |
|  | 12-oxo-LTB4 | 2.168152169 | 0.293833429 | 0.039222249 |
|  | L-Phenylalanine | 1.88912314 | 1.495953523 | 0.047087767 |
| MG | 12(R)-HETE | 21.77358455 | 0.149728377 | 2.54368E-06 |
|  | 4-Aminobenzoate | 5.142000269 | 57.63868377 | 7.13388E-06 |
|  | 20-Hydroxyarachidonic acid | 5.750230923 | 0.154904365 | 3.93934E-05 |
|  | 20-HETE | 1.887379495 | 0.045024694 | 4.64342E-05 |
|  | Sphinganine | 1.333298963 | 1.441483873 | 0.000490866 |
|  | Dihydrothymine | 2.059880729 | 0.539772567 | 0.000551341 |
|  | 12-oxo-ETE | 1.50359136 | 0.175678069 | 0.000920561 |
|  | m-Chlorohippuric acid | 1.037127454 | 2.257082094 | 0.001175318 |
|  | 5(S)-HETE | 13.10277901 | 0.108733818 | 0.001249784 |
|  | D-Lactose | 1.835366949 | 3.392981559 | 0.001822184 |
|  | Palmitic acid | 6.449318676 | 0.853228223 | 0.002025357 |
|  | Thioetheramide-PC | 11.58751478 | 1.739763986 | 0.002219127 |
|  | O-Acetyl-L-serine | 1.51386173 | 0.650357199 | 0.003254251 |
|  | L-Leucine | 1.74459311 | 1.416128723 | 0.004038156 |
|  | 8(S)-HETE | 1.561317363 | 0.255753862 | 0.005139868 |
|  | 2-hydroxy-butanoic acid | 2.340453899 | 4.146446744 | 0.006081579 |
|  | L-Carnitine | 1.620397282 | 0.693312873 | 0.006657962 |
|  | Cyclohexylamine | 1.31546264 | 0.493060559 | 0.006687185 |
|  | alpha-Tocopherol (Vitamin E) | 1.16513791 | 0.336918147 | 0.006955511 |
|  | LTB4 | 3.176417558 | 0.126442426 | 0.00860033 |
|  | (3-Carboxypropyl)trimethylammonium cation | 8.124436922 | 0.471123696 | 0.009167789 |
|  | Maltotriose | 2.626763157 | 8.831735965 | 0.011567672 |
|  | 12-oxo-LTB4 | 2.260814276 | 0.257454932 | 0.013161026 |
|  | 3.alpha.-Mannobiose | 2.731445462 | 3.097557736 | 0.014196314 |
|  | 1-Methylhistidine | 1.112536187 | 0.330105835 | 0.01506972 |
|  | Cholesteryl sulfate | 2.174734348 | 0.648232443 | 0.015447888 |
|  | 2-Oleoyl-1-stearoyl-sn-glycero-3-phosphoserine | 1.141199277 | 0.655050715 | 0.017261937 |
|  | Isomaltose | 2.599345197 | 3.096503514 | 0.017781076 |
|  | Hydroxyisocaproic acid | 2.395973027 | 5.982411655 | 0.019342402 |
|  | Pantothenate | 2.395644557 | 0.625293399 | 0.019592061 |
|  | 5(S)-HpETE | 2.073628839 | 0.250075244 | 0.022031312 |
|  | L-Gulonic gamma-lactone | 1.041466832 | 2.11220842 | 0.026929607 |
|  | sn-Glycerol 3-phosphoethanolamine | 2.265674264 | 1.652763204 | 0.030927592 |
|  | 2-Oxoadipic acid | 5.606863468 | 1.354882966 | 0.037972705 |
|  | PC(16:0/16:0) | 4.027885724 | 1.75670553 | 0.04065418 |
|  | Nervonic acid | 2.578030712 | 0.773030851 | 0.049056883 |
| PD | Salidroside | 1.389831171 | 57.26671214 | 1.37596E-05 |
|  | 12(R)-HETE | 11.13006015 | 1.426383666 | 0.012149808 |
|  | 20-HETE | 1.499423678 | 2.090719747 | 0.01511405 |
|  | D-Aspartic acid | 1.436966311 | 0.626575225 | 0.015283285 |
|  | 1,2-Benzenedicarboxylic acid | 1.416819225 | 0.758300938 | 0.016348927 |
|  | Hydroxyisocaproic acid | 2.48770504 | 7.583623501 | 0.016936995 |
|  | 2-Oleoyl-1-stearoyl-sn-glycero-3-phosphoserine | 1.274364308 | 0.569962772 | 0.022598562 |
|  | N-Tetracosanoyl-4-sphingenyl-1-O-phosphorylcholine | 1.27936456 | 0.517795007 | 0.023834728 |
|  | 20-Hydroxyarachidonic acid | 3.810231585 | 1.417364455 | 0.024935974 |
|  | Erucamide | 1.713121464 | 0.44365599 | 0.027806298 |
|  | 2-hydroxy-butanoic acid | 1.645378091 | 3.274502188 | 0.033149338 |
|  | Linoleic acid | 1.551393605 | 2.81340892 | 0.033245497 |
|  | D-Mannose | 2.005048518 | 1.637058256 | 0.038688696 |
|  | N-Palmitoylsphingosine | 1.270981643 | 0.565684704 | 0.042891082 |
|  | Thioetheramide-PC | 8.307470048 | 1.475276013 | 0.048774932 |
| PG | Salidroside | 1.949044714 | 80.375905 | 7.30556E-07 |
|  | Xanthine | 1.401722863 | 5.906240205 | 1.2624E-05 |
|  | Penicillic Acid | 1.457148397 | 6.260244994 | 3.25546E-05 |
|  | Phenol | 1.221337902 | 8.610582227 | 5.26684E-05 |
|  | Succinate | 1.671208993 | 0.505051478 | 0.003481582 |
|  | Palmitic acid | 9.28616091 | 0.900318343 | 0.00431458 |
|  | L-Carnitine | 2.019909696 | 0.715311359 | 0.004371574 |
|  | D-Proline | 1.670329425 | 1.285583333 | 0.009924001 |
|  | Methylmalonic acid | 2.169861291 | 0.539428469 | 0.019373055 |
|  | D-Aspartic acid | 1.672757352 | 0.728080198 | 0.022951309 |
|  | alpha-Tocopherol (Vitamin E) | 1.192924681 | 0.48195718 | 0.023349384 |
|  | L-Leucine | 1.643927973 | 1.299844993 | 0.037853435 |
|  | Adenosine | 3.72461241 | 0.501889186 | 0.04623846 |
|  | 2-Oxoadipic acid | 1.471648746 | 1.213902238 | 0.046856561 |

MD: MS-222 low-concentration group

MG: MS-222 high-concentration group

PD: 2-PE low-concentration group

PG: 2-PE high-concentration group

Table 2. Primers for genes used for quantitative reverse transcription PCR

| Primer Name | Sequence (5'to3') |
| --- | --- |
| CI01113194_00000034_00007377-F | TCGCTGGAGGCAAAGATCAGC |
| CI01113194_00000034_00007377-R | GCCCCAAATCCACTGAGCCA |
| CI01000168_01110611_01115503-F | TACACCGACACGCTCCCTGA |
| CI01000168_01110611_01115503-R | CTCGGCTCTTCAGTGCTGCT |
| CI01000325_05510163_05515246-F | TGAGTCCCTGTTTTCGGTGGA |
| CI01000325_05510163_05515246-R | ATCCTGGGGTGGGCTTCTGT |
| CI01000426_00021251_00036674-F | ACCGTTTCCCATCTACACAGCG |
| CI01000426_00021251_00036674-R | TTGCTGCCAAAGCTGGAGGT |
| CI01000170_00049973_00068447-F | CATGGTGGGTTTGCTGGGAT |
| CI01000170_00049973_00068447-R | TCCAGACACTCCGCTCAGAT |
| CI01000330_03692275_03699509-F | TGGGCGTTCCTCGTGTTTGG |
| CI01000330_03692275_03699509-R | GACCAAGCTGTCCGCAAGAGT |
| CI01000339_01860747_01871902-F | GAGCGAGTGGGAGGAGCTTT |
| CI01000339_01860747_01871902-R | TTCTCTTTAGTTTGAAGGCGTCTGT |
| CI01000055_01852604_01874289-F | GCTGGCAATAACACAGACGGC |
| CI01000055_01852604_01874289-R | GCAGAAGGCACTGGGGGAAA |
